# Convergent Evolution among Ruminant-Pathogenic *Mycoplasma* Involved Extensive Gene Content Changes

**DOI:** 10.1093/gbe/evy172

**Published:** 2018-08-08

**Authors:** Wen-Sui Lo, Gail E Gasparich, Chih-Horng Kuo

**Affiliations:** 1 Institute of Plant and Microbial Biology, Academia Sinica, Taipei, Taiwan; 2 College of Arts and Sciences, Salem State University; 3 Department for Evolutionary Biology, Max-Planck Institute for Developmental Biology, Tuebingen, Germany

**Keywords:** *Mycoplasma*, Entomoplasmataceae, Mollicutes, genome evolution, pathogen, gene transfer

## Abstract

Convergent evolution, a process by which organisms evolved independently to have similar traits, provides opportunities to understand adaptation. The bacterial genus *Mycoplasma* contains multiple species that evolved independently to become ruminant pathogens, which represents an interesting study system for investigating the process. In this work, we determined the genome sequences of 11 *Entomoplasma*/*Mesoplasma* species. This new data set, together with the other available Mollicutes genomes, provided comprehensive taxon sampling for inferring the gene content evolution that led to the emergence of *Mycoplasma* Mycoides cluster. Our results indicated that the most recent common ancestor (MRCA) of the Mycoides-Entomoplasmataceae clade lost ∼15% of the core genes when it diverged from the *Spiroplasma* Apis clade. After this initial wave of genome reduction, relatively few gene gains or losses were inferred until the emergence of the Mycoides cluster. Compared with those Entomoplasmataceae lineages that maintained the association with insects, the MRCA of the Mycoides cluster experienced a second wave of gene losses, as well as acquiring >100 novel genes through horizontal gene transfer. These gene acquisitions involved many with the *Mycoplasma* Hominis/Pneumoniae lineages as the putative donors, suggesting that gene exchanges among these vertebrate symbionts with distinct phylogenetic affiliations may be important in the emergence of the Mycoides cluster. These findings demonstrated that the gene content of bacterial genomes could be exceedingly dynamic, even for those symbionts with highly reduced genomes. Moreover, the emergence of novel pathogens may involve extensive remodeling of gene content, rather than acquisition of few virulence genes.

## Introduction

Convergent evolution, a process by which different organisms evolved to have similar traits independently, often offers excellent opportunities to understand adaptation. For example, when bacteria with different ancestors evolved to become similar pathogens, comparative genomics among these lineages could provide insights into the genetic mechanisms for transitions between ecological niches. The bacterial genus *Mycoplasma* is a good study system for such investigations. Despite the similarity in cell morphology and ecological niches, the species in this polyphyletic genus belong to three distinct clades, namely Mycoides, Hominis, and Pneumoniae (Brown 2010). The Mycoides cluster emerged from a diverse group that are mostly commensals of insects, including the polyphyletic *Entomoplasma*/*Mesoplasma* and the paraphyletic *Spiroplasma*, and became pathogens of ruminants that are capable of direct transmission without insect vectors (Gasparich 2004). The few recognized species within the Mycoides cluster include *Mycoplasma mycoides*, which is the type species of the genus, as well as that of the family Mycoplasmataceae and the order Mycoplasmatales (Brown 2010). However, most of the >100 *Mycoplasma* species belong to the other two clades, which diverged from the Mycoides cluster prior to the diversification of the *Spiroplasma*–Entomoplasmataceae–Mycoides clade. In other words, the Mycoides clade emerged from an insect-associated ancestor and evolved to become phenotypically similar with other *Mycoplasma* lineages through independent events.

While the entangled evolutionary relationships and taxonomy of the aforementioned bacteria often create confusion, the diversity also provides opportunity for investigating symbiont evolution. For instance, the changes in ecological niches (i.e., from insect commensals to vertebrate pathogens) and cell morphology (i.e., from helical to coccoid) raised the question of what the underlying genetic differentiations for these phenotypic variations are. Moreover, if acquisition of novel genes was involved, more detailed investigation of these horizontal gene transfer (HGT) events (e.g., identification of the donors) could provide further insights into the processes of pathogen evolution.

However, addressing these questions was difficult due to the high levels of phylogenetic divergence and genetic differentiations between the Mycoides cluster and other relevant lineages with high quality genomic information available (e.g., *Mycoplasma* species in the Hominis/Pneumoniae groups and *Spiroplasma*). To overcome this difficulty, we determined the genome sequences of 11 *Entomoplasma*/*Mesoplasma* species. This new data set, together with the other six *Mesoplasma* genomes that have been published recently (Mukherjee et al. 2017), encompasses all recognized species within the genus *Entomoplasma*/*Mesoplasma* (Gasparich 2014). These lineages in the family Entomoplasmataceae are the closest extant relatives of the Mycoides cluster species, such that the availability of these genomes allows for detailed inference of gene content evolution leading to the emergence of the Mycoides clade from an insect-associated ancestor. Moreover, the taxonomy of *Entomoplasma*/*Mesoplasma* is known to conflict with the 16S rDNA phylogeny (Johansson and Pettersson 2002; Gasparich 2004, 2014). With the genome-scale molecular phylogeny and gene content analysis, this study aims to resolve this long-standing issue.

## Materials and Methods

### Genome Sequencing

The type strains of six *Entomoplasma* species were provided by Gail Gasparich and the five *Mesoplasma* species were acquired from the American Type Culture Collection (table 1). The procedures for genome sequencing and analysis were based on those described in our previous studies (Chung et al. 2013; Lo et al. 2013). The bioinformatics tools were used with the default settings unless stated otherwise. Briefly, one paired-end library (insert size = ∼550 bp) was prepared for each sample and sequenced using the Illumina MiSeq platform (Illumina, USA). The average coverage was ∼880×. The de novo assembly was performed using Velvet v1.2.07 (Zerbino and Birney 2008) and iteratively improved by examining the raw read mapping results until the genome is complete or could not be improved further. The gene prediction was done using RNAmmer (Lagesen et al. 2007), tRNAscan-SE (Lowe and Eddy 1997), and Prodigal (Hyatt et al. 2010). The annotation was based on the homologous genes in published Mollicutes genomes (table 1) as identified by OrthoMCL (Li et al. 2003), followed by manual curation based on database searches (Clark et al. 2016; Kanehisa et al. 2016). For the OrthoMCL analysis, the e-value cutoff at the all-against-all BLASTP (Camacho et al. 2009) step was set to 1×e^−15^ and the inflation parameter at the Markov Clustering step was set to 1.5.

**Table 1:** Genome Characteristics of Representative Species in the Families Spiroplasmataceae, Entomoplasmataceae, and Mycoplasmataceae

Clade	**Species**^a^	Host	Accession	Genome Size (kb)	% GC	% Coding	Protein-Coding Genes	Pseudogenes	tRNA Genes	rRNA Genes
Citri-Chrysopicola-Mirum	*S. kunkelii*	Insect/Plant	NZ_CP010899	1,464	25.0	67.2	1,330	211	32	3
	*S. chrysopicola*	Insect	CP005077	1,123	28.8	89.0	1,009	6	33	3
	*S. eriocheiris*	Crustacean	CP011856	1,366	29.8	86.0	1,180	30	32	3
Apis	*S. apis*	Insect	CP006682	1,161	28.3	87.8	997	1	29	3
	*S. culicicola*	Insect	CP006681	1,175	26.4	92.2	1,071	0	29	3
	*S. sabaudiense*	Insect	CP006934	1,076	30.2	90.0	924	7	30	6
Lactucae	*Me. lactucae*^b^	Plant (surface)	CP024967	837	29.8	89.4	692	1	32	9
Lucivorax	*E. luminosum*^b^	Insect	CP024963	1,032	29.8	89.3	868	6	32	3
	*E. lucivorax*^b^	Insect	PHNE00000000	1,127	30.3	88.2	961	7	31	3
	*E. freundtii*^b^	Insect	CP024962	838	34.6	90.2	702	3	33	6
	*E. somnilux*^b^	Insect	CP024965	868	28.0	91.5	725	2	32	3
Seiffertii	*Me. seiffertii*	Plant (surface)	GCA_000518725	978	30.2	89.2	807	18	29	7
	*Me. syrphidae*	Insect	GCA_000686545	918	30.2	87.7	740	15	29	12
	*A. multilocale*	Horse/Rabbit	GCF_000483165	925	30.6	89.1	814	15	28	8
	*Me. photuris*	Insect	GCA_000702725	779	28.1	91.4	681	10	28	8
Florum	*Me. florum*	Plant (surface)	NC_006055	793	27.0	92.7	680	0	29	6
	*Me. grammopterae*	Insect	GCA_000701525	807	26.9	91.4	689	13	29	5
	*Me. entomophilum*^b^	Insect	CP024966	848	27.2	91.7	714	7	29	6
	*Me. coleopterae*^b^	Insect	CP024968	800	27.2	91.3	691	3	29	6
	*Me. tabanidae*^b^	Insect	CP024969	847	26.9	91.6	722	7	29	6
	*E. melaleucae*^b^	Plant (surface)	CP024964	845	26.6	80.3	789	57	29	6
	*Me. chauliocola*	Insect	GCA_000518825	844	26.9	87.6	710	25	29	7
	*E. ellychniae*^b^	Insect	PHND00000000	900	26.2	88.6	786	7	29	3
	*Me. corruscae*^b^	Insect	PHNF00000000	839	26.3	89.5	701	4	29	3
Mycoides	*My. mycoides*	Cattle/Goat	NC_005364	1,212	24.0	81.5	1,017	0	30	6
	*My. capricolum*	Goat	NC_007633	1,010	23.8	86.0	793	35	30	6
	*My. leachii*	Cattle	NC_014751	1,009	23.8	85.8	835	31	30	6
	*My. putrefaciens*	Goat	NC_015946	833	26.9	83.7	654	25	30	6
	*My. yeatsii*	Goat	NZ_CP007520	895	25.7	89.5	747	8	30	6
Hominis	*My. agalactiae*	Goat	NC_009497	877	29.7	79.6	666	63	34	6
	*My. hyopneumoniae*	Pig	NC_007295	897	28.5	85.5	665	18	30	3
	*My. mobile*	Fish	NC_006908	777	25.0	90.4	649	6	28	3
Pneumoniae	*My. penetrans*	Human	NC_004432	1,359	25.7	87.0	1,022	10	30	3
	*My. genitalium*	Human	NC_000908	580	31.7	93.7	507	7	36	3
	*My. gallisepticum*	Bird	NC_004829	1,013	31.5	86.5	747	24	32	6

Note.—All six species in the genus *Entomoplasma* and all 11 species in *Mesoplasma* are included.

a Genus name abbreviations: *A*., *Acholeplasma*; *E*., *Entomoplasma*; *Me*., *Mesoplasma*; *My*., *Mycoplasma*; *S*., *Spiroplasma*.

b Genome sequences newly reported in this study.

### Phylogenetic Analysis

The procedure for phylogenetic inference was based on that described in our previous studies (Chung et al. 2013; Lo et al. 2013; Lo and Kuo 2017). Briefly, the amino acid sequences of conserved single-copy genes were aligned using MUSCLE v3.8 (Edgar 2004). The concatenated alignment was analyzed using PhyML v3.0 (Guindon and Gascuel 2003); the proportion of invariable sites and the gamma distribution parameter were estimated from the data set and the number of substitute rate categories was set to four. The bootstrap supports were estimated based on 1,000 replicates, the rooting was based on a class-level 16S rDNA phylogeny of Mollicutes (Gasparich 2004). To validate the maximum likelihood phylogeny, we performed Bayesian inference using MrBayes v3.1.2 (Ronquist and Huelsenbeck 2003). The amino acid substitution model was set to mixed with gamma-distributed rate variation across sites and a proportion of invariable sites. The number of rate categories for the gamma distribution was set to four. The Markov chain Monte Carlo analysis was set to run for 1,000,000 generations and sampled every 100 generations. The first 25% of the samples were discarded as the burn-in.

### Gene Content Analysis

The homologous gene clustering result produced by OrthoMCL was converted into a matrix of 35 species × 5,752 gene clusters; the value in each cell indicates the gene copy number. This matrix was imported into R to perform two statistical analyses of gene content dissimilarity. For hierarchical clustering, we used the PVCLUST package (Suzuki and Shimodaira 2006); the clustering support was based on the approximately unbiased *P*-values as suggested by the authors. For principal coordinates analysis, we used the PCOA function in the APE package (Popescu et al. 2012) based on the Jaccard distance matrix calculated using the VEGAN package in R.

### Inference of Gene Content Evolution

To infer the ancestral state of gene content in each major clade, we assigned gene presence when >70% of the extant species containing the homologous gene in question or absence when <30% of the extant species containing the gene. The possible events of gene gains/losses were inferred manually from the patterns of presence/absence based on the parsimony principle. For cases that the number of extant species containing the gene falls in between these cutoff values, the ancestral state was undefined and the inference of gains/losses was not performed. More detailed information is provided in supplementary fig. S1, Supplementary Material online.

To identify the horizontally acquired gene islands in the Mycoides clade, we chose *My. mycoides* as the representative. The putatively acquired genes (i.e., the genes gained along the internal branches “IV” and “VI” in fig. 3, as well as the terminal branch leading to *My. mycoides*) were used as the queries to run BLASTP (Camacho et al. 2009) searches against the NCBI nonredundant database (Clark et al. 2016) (e-value cutoff = 1 ×10^−^^15^; max target sequences = 2,000). A candidate was defined as acquired if after excluding hits from Mycoides clade, the best hit was from outside of the *Spiroplasma*-Entomoplasmataceae-Mycoides clade. A chromosomal segment was identified as an acquired island if it contains at least five acquired genes. Up to three nonacquired genes was allowed in between pairs of acquired genes when defining islands to accommodate disruption of gene order by biological processes (e.g., genome rearrangement) or artifacts (e.g., unidentified pseudogene fragments or errors in genes prediction). To investigate the distribution of these islands in the *Mycoplasma* Hominis/Pneumoniae clades, *Mycoplasma/Ureaplasma* species that shared at least one island with *My. mycoides* and all five Mycoides clade species were included in a second run of homologous gene cluster identification by OrthoMCL. The result was examined to identify the acquired genes shared (supplementary table S2, Supplementary Material online) and synteny conservation.

## Results

### Genome Overview, Phylogeny, and Gene Content

The genome characteristics and hosts of those 35 species compared in this study are summarized in table 1. Representatives of the class Mollicutes are known for their reduced genomes and biased nucleotide compositions; most species have a chromosome that is ∼1,000 kb in size or smaller and an overall G + C content of ∼<30%.

**Fig. 1.**
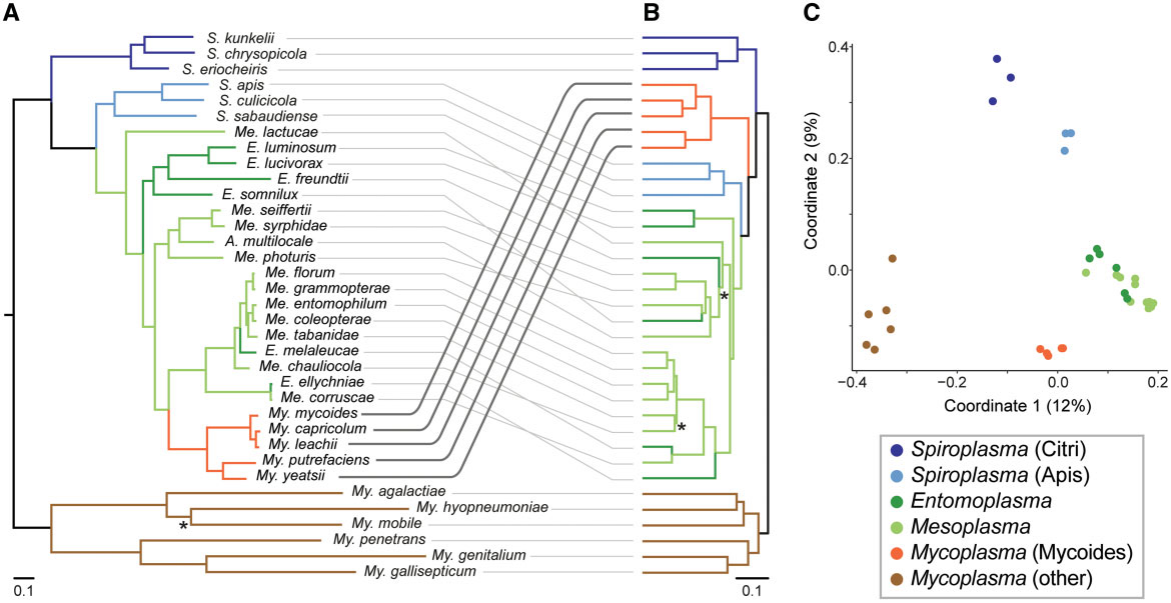
—Molecular phylogeny and gene content dissimilarity of the Mycoplasmatales–Entomoplasmatales clade lineages. (*A*) Maximum likelihood phylogeny based on 161 shared single-copy genes (71,550 aligned amino acid sites). One node with <98% bootstrap support is labeled with “*”. Bayesian inference based on the same concatenated alignment produced a phylogeny with identical topology and comparable levels of support. (*B*) Hierarchical clustering based on gene content. Two nodes with <80% support are labeled with “*”. Note the shift in position of the Mycoides clade lineages. (*C*) Principal coordinates analysis of gene content. The % variance explained by each axis is provided in parentheses.

Among these 35 representative species, we identified 5,752 homologous gene clusters. Based on a concatenated protein alignment of the 161 conserved single-copy genes (supplementary table S1, Supplementary Material online), we inferred a maximum likelihood phylogeny (fig. 1*A*) that is largely consistent with the previous results based on 16S rDNA (Gasparich 2004, 2014; Volokhov et al. 2012) and *rpoB* (Volokhov et al. 2012) but with better resolution and stronger support. Moreover, validation by using the Bayesian method (Ronquist and Huelsenbeck 2003) produced a phylogeny that has an identical topology and comparable levels of support with the maximum likelihood result. All five *Mycoplasma* species belonging to the Mycoides cluster form a monophyletic clade with 100% bootstrap support, while the genera *Entomoplasma*/*Mesoplasma* are both polyphyletic and have entangled relationships. Based on the phylogeny, lineages in these two genera could be classified into four major clades (fig. 1*A* and table 1). Notably, the species *Acholeplasma multilocale* (Hill et al. 1992) was found to be affiliated with Entomoplasmataceae rather than other *Acholeplasma* species. This finding is consistent with previous studies based on single-locus molecular phylogeny (Volokhov et al. 2007, 2012) and metabolic profiling (Pollack et al. 1996), thus providing further support for this species to be re-classified as *Mesoplasma* following other precedents (Tully et al. 1993).

The hierarchical clustering of gene content dissimilarity (fig. 1*B*) produced a pattern that is similar to the molecular phylogeny (fig. 1*A*). This result is expected when the history of gene content evolution was dominated by vertical inheritance, such that closely related species are similar in their gene content. However, one major exception is that the Mycoides cluster became sister to the *Spiroplasma* Apis clade and Entomoplasmataceae, rather than being nested within Entomoplasmataceae as expected based on phylogeny. This finding indicated that the gene content of those Mycoides cluster lineages diverged substantially from their sisters in Entomoplasmataceae. To provide an alternative method of data visualization, as well as to investigate the directionality of gene content change, we conducted a principal coordinates analysis (fig. 1*C*). This analysis presents the differences in gene content as distances in a two-dimensional space. Based on this plot, each of the major groups in current taxonomy (Tully et al. 1993; Brown 2010) represents a cohesive collection of extant species that are similar in their gene content. For example, despite the phylogenetic divergence among those Entomoplasmataceae species assigned to either *Entomoplasma* or *Mesoplasma*, those species are still highly similar in their gene content while distinct from other Mollicutes. Similarly, despite the even higher levels of sequence divergence observed among those six representative *Mycoplasma* species belonging to the Hominis/Pneumoniae groups (fig. 1*A*), all those six species form a cohesive and distinct group based on gene content (fig. 1*C*). Intriguingly, based on this principal coordinates analysis, those Mycoides cluster species not only diverged from their Entomoplasmataceae sisters, but also became more similar to other *Mycoplasma* species in their gene content (fig. 1*C*). This pattern suggests that HGT may have played an important role in the emergence of the Mycoides cluster. However, one caveat of this analysis is that by visualizing the gene content differences in a two-dimensional space, it is possible to lose some signals in the original data set. In this particular case, the proportion of variance explained by the first two coordinates are relatively low (12% for PC1 and 9% for PC2). Therefore, this analysis is suitable for providing a visual summary and must be complemented by other in-depth analyses.

### Gene Content of Extant Species

**Fig. 2.**
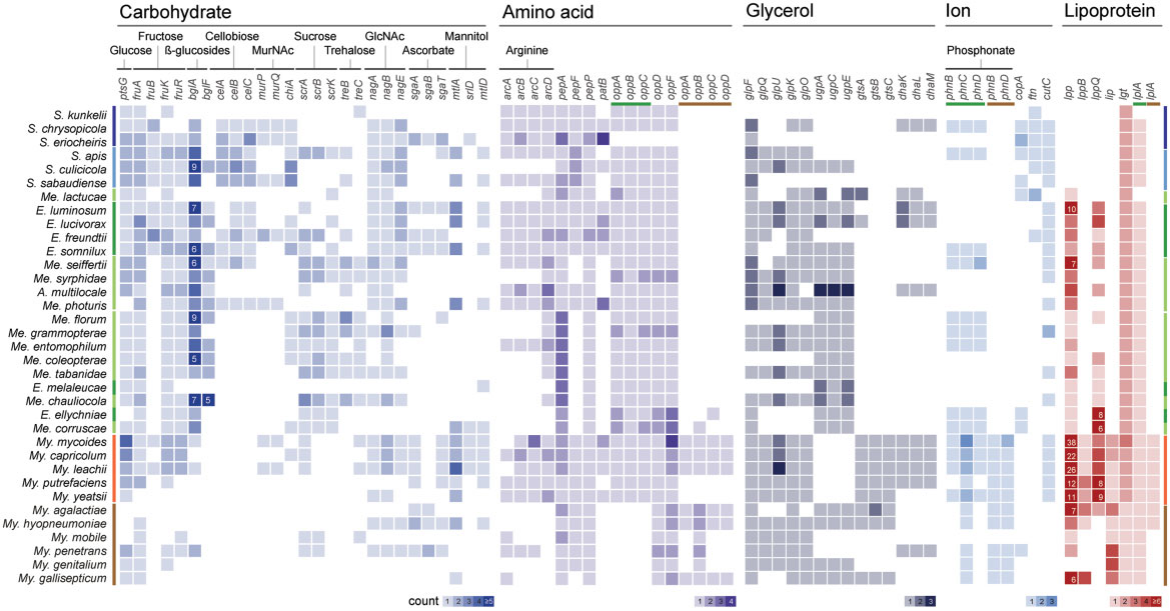
—Presence and absence of genes related to substrate transport, metabolism, and pathogenicity. Three sets of genes could be classified as two distinct types based on sequence similarities among homologous genes (i.e., *oppA/B/C/D*, *phnB/D*, and *lplA*) and the gene names are color-coded (green, Entomoplasmatales-type; brown, Mycoplasmatales-type).

For more detailed examination of the gene content, we identified the patterns of presence and absence for homologous genes involved in major metabolic pathways (fig. 2). One notable finding is that the Mycoides clade lineages have two sets of functional homologs for several genes, one inherited vertically from their *Spiroplasma*-Entomoplasmataceae ancestor and the other acquired horizontally from other more divergent *Mycoplasma* lineages. Examples include oligopeptide transporter (*oppA*/*B*/*C*/*D*), phosphonate transporter (*phnB*/*D*), and lipoate protein ligase A (*lplA*).

The glycerol metabolism has been demonstrated as critical for *Mycoplasma* pathogenicity (Pilo et al. 2005, 2007; Hames et al. 2009). The main virulence gene *glpO* appeared to be present in the MRCA of these Mollicutes lineages. Consistent with the previous finding that this gene had been lost independently multiple times among various *Spiroplasma* lineages (Chang et al. 2014; Lo and Kuo 2017), here we found that several *Entomoplasma*/*Mesoplasma* species lack this gene as well. This finding suggested that there was no strong selection pressure for maintaining this gene in these insect symbionts.

The current Mollicutes taxonomy was last revised in 1993 (Tully et al. 1993). Within the family Entomoplasmataceae, the requirement of sterol for growth is the defining character for *Entomoplasma*, whereas the nonsterol-requiring species are assigned to *Mesoplasma*. Previous studies have indicated that the sterol requirement is not a phylogenetically informative character (Johansson and Pettersson 2002; Gasparich 2004). In our gene content analysis, we did not find any homologous gene cluster that is present in all species of one genus and absent in the other. Moreover, we did not find any obvious candidate genes that may be linked to the sterol requirement. Although it is possible that these findings may be due to artifacts of gene prediction and annotation, or the limitation that eight of these 17 *Entomoplasma*/*Mesoplasma* genomes were incomplete, it is also possible that this phenotypic difference is due to gene expression regulation rather than gene presence/absence. In support of this possibility, we found that the patterns of presence/absence for arginine metabolism genes (*arcA/B/C/D*) do not match the phenotyping results perfectly. Among the 17 *Entomoplasma*/*Mesoplasma* species, only *Entomoplasma freundtii* and *Mesoplasma photuris* were found to be capable of hydrolyzing arginine (Gasparich 2014). While we found the *arcA/B/C/D* genes in these two species, several other species have these genes as well.

### Putative Gene Gains and Losses

**Fig. 3.**
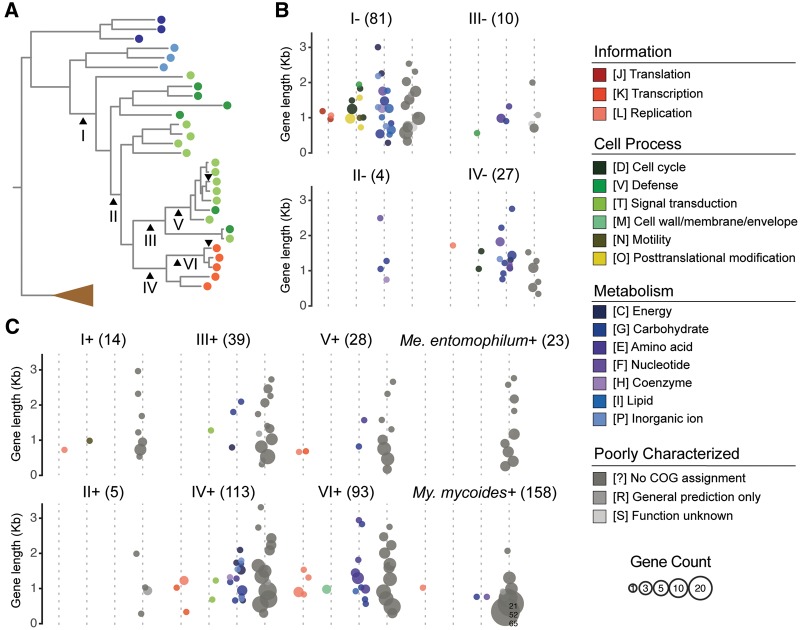
—Characteristics of the genes gained and lost. (*A*) Phylogenetic positions of the six internal branches and two terminal branches analyzed in this study. The species phylogeny is based on the result from figure 1*A*. (*B*) Putative gene losses. The number in parentheses above each subpanel indicates the total number of inferred gene losses. The genes are binned by length using 250-bp intervals and color-coded according to their functional category assignments. The circle size is proportional to the number of genes in each bin. (*C*) Putative gene gains.

Based on the molecular phylogeny and the gene content of extant species, we further inferred the putative gene gains and losses for six internal branches and two terminal branches (fig. 3; supplementary table S1, Supplementary Material online). For branch “I”, in which the Mycoides-Entomoplasmataceae clade diverged from their *Spiroplasma* ancestor, we inferred 81 putative losses and 14 putative gains. Because the Lactucae clade has only one extant species (i.e., *Mesoplasma lactucae*) with a long terminal branch, the gene presence/absence in this species was ignored in the initial inference. Nonetheless, we found that the gene presence/absence in this species is consistent for 75/81 (93%) of the inferred losses and 11/14 (79%) of the inferred gains. The few discrepancies may be explained by either gene gains/losses occurred in the terminal branch leading to *Me. lactucae*, or the gains/losses actually occurred in the internal branch between “I” and “II”. Regardless which scenario was true, these inferred gains and losses are applicable to most, if not all, extant species of the Mycoides-Entomoplasmataceae clade. Considering that 531 homologous gene clusters are shared by the *Spiroplasma* Apis and Citri clades, the loss of these 81 genes represents a 15% reduction in the core genome. The majority of these losses are related to metabolism, such as those related to the iron-sulfur (Fe-S) clusters production (*sufB*/*C*/*D*), formate metabolism (*fhs* and *pflA*/*D*), and lipids synthesis (*dxr*, *dxs*, and *ispD*/*E*/*F*/*G*/*H*). Additionally, genes related to the helical morphology of *Spiroplasma* (*fib* and *mreB*) were also lost, which is consistent with the coccoid cell shape of the Mycoides-Entomoplasmataceae clade species. Compared with gene losses, the putative gene gains are fewer in number. It is possible that most gene gains did not contribute to fitness, thus were not preserved by selection (Kuo and Ochman 2009a). In this regard, it is interesting to note that most of the 14 ancient gene gains that have been preserved do not have specific functional annotation. The explanation for this observation is unclear.

After the initial wave of genome reduction occurred near the root of the Mycoides-Entomoplasmataceae clade, relatively few gains and losses were inferred until the emergence of the Mycoides clade (fig. 3; supplementary table S1, Supplementary Material online). For the internal branch between “II” and “III”, only two losses were inferred and no gain was inferred. The gene losses in “V” and “VI” could not be inferred confidently given the available taxon sampling, so we chose to omit this part. By comparing the patterns of gene content evolution between the Mycoides clade and its sister group that maintained the ancestral state of being insect commensals, we found many more gene acquisitions in the lineages that evolved to become pathogens of ruminants (e.g., branches “IV” and “VI”). Although many of these novel genes encode short hypothetical proteins and may be in fact fragments of unannotated pseudogenes, >20% have specific functional annotation and identifiable homologs in the more divergent *Mycoplasma* species belonging to the Hominis and Pneumoniae groups.

### Gene Gains in Mycoides Cluster Species

**Fig. 4.**
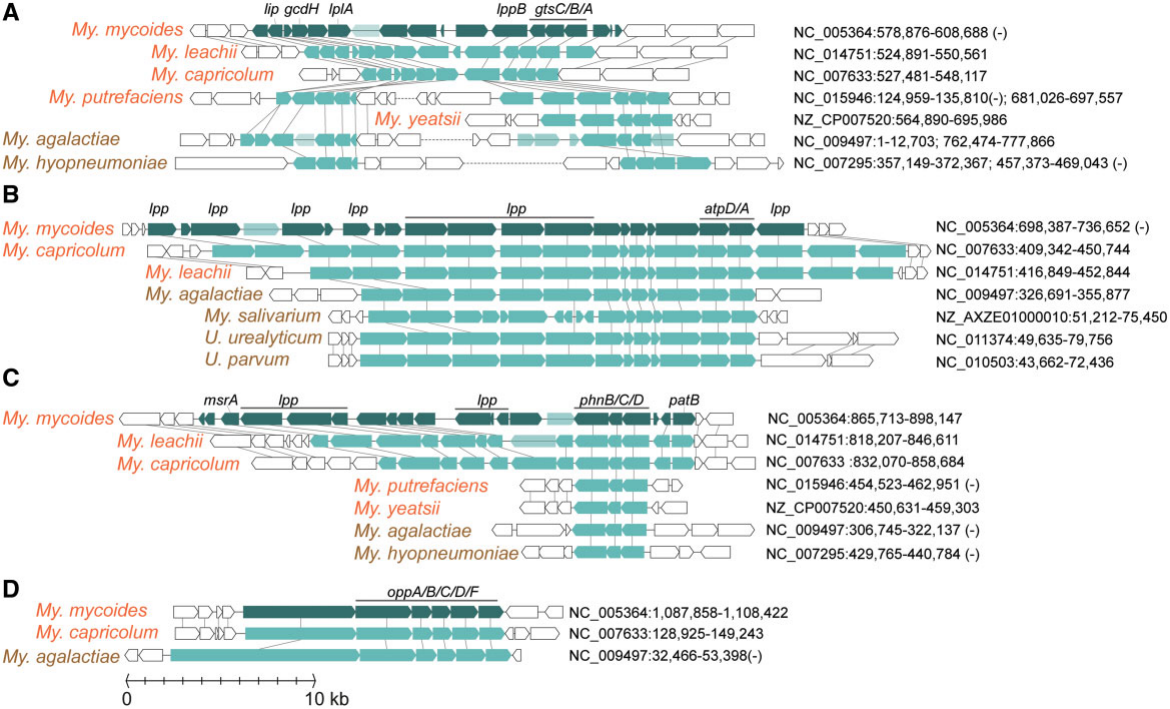
—Organization of horizontally acquired gene islands in *Mycoplasma mycoides* (dark green) and their homologous genes in other genomes (light green). Annotated pseudogenes are indicated by transparent light green; neighbor genes that were not involved in horizontal transfer are shown in white. The sequence accession numbers and start/end positions of these regions are labeled.

Using *My. mycoides* as the representative of the Mycoides cluster, we identified a total of 409 genes belonging to 364 homologous gene clusters as putatively acquired, which are distributed across 31 islands on the chromosome. Our search for potential donors of these genes identified 37 representative Mycoplasmatales species outside of the Mycoides cluster (supplementary table S2, Supplementary Material online). Despite the high levels of genetic divergence among these lineages, several sites of micro synteny conservation were found (fig. 4). Three of these acquired islands contain multiple lipoprotein genes (*lpp* and *lppB*), which are known to play central roles in *Mycoplasma* pathogenesis (Browning et al. 2011). These gene acquisitions contributed to the extensive *lpp* copy number expansion observed among the Mycoides cluster species (fig. 2). Additionally, the gain of a *oppA*/*B*/*C*/*D*/*F* gene cluster not only provided a second set of genes for oligopeptide transporter (fig. 2), but may also facilitate cytoadherence to host cells (Henrich et al. 1999; Distelhorst et al. 2017). Finally, the gain of a glycerol transporter gene cluster (*gtsA*/*B*/*C*) may compensate the Mycoides-specific loss of sn-glycerol-3-phosphate transporter (*ugpA*/*C*/*E*) (figs. 2 and 4*A*).

## Discussion

### Insights into *Mycoplasma* Evolution

Based on our findings, the emergence of the Mycoides cluster involved massive changes in gene content through losses and gains. The elevated levels of genetic drift in symbionts (Kuo et al. 2009), combined with the deletional bias commonly observed in bacterial genomes (Mira et al. 2001; Kuo and Ochman 2009b), have been linked to genome reduction (Moran 2002; McCutcheon and Moran 2012; Lo et al. 2016). Intriguingly, in addition to the gene losses commonly observed in symbiont genome evolution, here we also observed extensive gene gains, which was thought to be rare in obligate pathogens with small genomes (Ochman and Davalos 2006).

Mycoplasmas, particularly those Mycoides clade lineages, were known for their genome plasticity (Thiaucourt et al. 2011). Mixed infection in the same host by divergent species and the presence of integrative conjugative elements have been proposed as the major factors for their frequent genetic exchanges (Citti and Blanchard 2013; Citti et al. 2018). In an early genomic characterization of *Mycoplasma agalactiae* in the Hominis group, ∼18% of the genome was proposed as putatively acquired by HGT from the Mycoides cluster (Sirand-Pugnet et al. 2007). However, with the improvement in taxon sampling of available genomes, these genes that are shared between few Mycoides and Hominis lineages appeared to be absent in Entomoplasmataceae and Spiroplasmataceae. Thus, the true origins of these genes, as well as the directionality of HGT, would require further investigation. Nevertheless, gene acquisitions appeared to have played an important role in promoting convergent evolution among divergent lineages within the genus *Mycoplasma*.

### Implications on Mollicutes Taxonomy

The comparison between molecular phylogeny and gene content divergence (fig. 1) provided several implications regarding Mollicutes phylogeny. Despite the phylogenetic divergence, all extant *Entomoplasma*/*Mesoplasma* species share similar gene content, which possibly reflects their similarity in ecological niches as insect symbionts (Gasparich 2014). This finding provided strong support to the previous proposals of merging *Mesoplasma* to *Entomoplasma* (Johansson and Pettersson 2002; Gasparich 2004). Furthermore, the availability of these genome sequences would allow for the adoption of a recent proposal on standardizing bacterial taxonomy based on genome-scale phylogeny (Parks et al. 2018). Based on the high similarities in chromosome organization, gene content, and sequences of shared genes, some of the extant species may be merged as well (e.g., *Mesoplasma florum* and *Mesoplasma grammopterae*, *Entomoplasma ellychniae*, *Mesoplasma corruscae*, etc.). With these proposed updates, the genus *Entomoplasma* would be paraphyletic, which is similar to the situation for *Spiroplasma* (Gasparich 2004). Unfortunately, no straightforward solution is available to resolve these conflicts between phylogeny and taxonomy (Gasparich 2004).

The issue regarding *Mycoplasma* taxonomy is more complex. The polyphyly of this genus has been known for decades and has attracted much attention (Weisburg et al. 1989; Tully et al. 1993; Gasparich 2004; Volokhov et al. 2012). One possible solution to reconcile phylogeny and taxonomy would be to reassign extant *Mycoplasma* species in the three major clades (i.e., Mycoides, Hominis, and Pneumoniae) to separate genera and families, such as those created in a recent proposal (Gupta et al. 2018). However, because these bacteria include many important pathogens of humans and domestic animals, the existing species names are associated with a large body of literature and government regulations. Therefore, any change to the taxonomy of this group would need to be considered carefully according to the International Code of Nomenclature of Prokaryotes (Parker et al. 2015).

## Conclusions

The genome sequences newly generated from this work with comprehensive taxon sampling in an important branch of Mollicutes provide valuable resources to the research community. In addition to informing taxonomy revisions, these data sets also provide a strong foundation for future investigation on the biology of these bacteria. Particularly, with the key roles that these Mollicutes species played in synthetic biology (Hutchison et al. 2016; Baby et al. 2018), these data sets could further facilitate the development of this field.

By adopting a phylogenetic framework to infer the genome evolution events that corresponded to the transition from insect symbionts to ruminant pathogens, this study illustrated the flexibility of Mollicutes genomes. Although HGT in diverse bacteria (Dagan et al. 2008; Vos et al. 2015) and genome reduction in pathogens (Moran 2002; Ochman and Davalos 2006; Weinert and Welch 2017) have been well-studied, *Mycoplasma* appeared to stand out among obligate pathogens with small genomes in their extreme genome plasticity. Such extensive HGT for re-shaping the gene content has been reported previously for *Spiroplasma* (Lo et al. 2015), and the shared ecological niches as insect symbionts may have promoted the HGT between *Spiroplasma* and *Entomoplasma*/*Mesoplasma* (Lo and Kuo 2017; Tsai et al. 2018). In this work, the extensive HGT between the Mycoides cluster and the other ruminant-pathogenic *Mycoplasma* that are phylogenetically divergent further highlighted the importance of shared ecological niches in facilitating HGT. Additionally, the same alternative genetic code shared among these Mollicutes species could also play an important role in the successful integration of acquired genes (Bové 1993; Sirand-Pugnet et al. 2007; Lo and Kuo 2017). In summary, with the combination of vertically inherited genes and horizontally acquired genes, these Mycoides clade lineages are effectively hybrids. For future studies, the generality of HGT as an evolutionary process for promoting convergent evolution remains to be investigated.

## Acknowledgments

The funding for this project was provided by the Institute of Plant and Microbial Biology at Academia Sinica to C.H.K. The funder had no role in study design, data collection and interpretation, or the decision to submit the work for publication. The *Mesoplasma* strains were imported under the permit number 103-B-002 issued by the Council of Agriculture of Taiwan. The Sanger sequencing service and the Illumina sequencing library preparation service was provided by the Genomic Technology Core (Institute of Plant and Microbial Biology, Academia Sinica). The Illumina MiSeq sequencing service was provided by the Genomics Core (Institute of Molecular Biology, Academia Sinica).

## Supplementary Material

Supplementary Data
