# Supplementary material for: Convergent Evolution among Ruminant-Pathogenic Mycoplasma Involved Extensive Gene Content Changes
Source: Genome Biol Evol. 2018 Aug 8;10(8):2130–9. doi: 10.1093/gbe/evy172 (PMC6117150; doi:10.1093/gbe/evy172)
Supplement: Supplementary Data [file evy172_supp.zip › Supp.docx]

**Supplementary Material**

Fig. S1. The criteria for inferring putative gene gains and losses. To infer the ancestral state of gene content in each major clade, we assigned presence when >70% of the extant species containing the homologous gene in question (i.e., allowing for few losses after the species divergence). Alternatively, we assigned absence in the ancestral state when <30% of the extant species containing the gene (i.e., allowing for few gene acquisitions). The exact numbers of extant species with or without the homologous gene are rounded to the nearest integers and shown in the figure. For cases that the number of extant species with the gene falls in between these cutoff values, the ancestral state was undefined and the inference of gains/losses was not performed. (A) Inference of ancient events close to the initial divergence of the Mycoides-Entomoplasmataceae clade, for which the patterns of gene presence/absence in *Spiroplasma* were considered. (B) Inference of more recent events, for which *Spiroplasma* and *Mesoplasma lactucae* were not considered.

Table S1. Lists of homologous gene clusters.

Table S2. Number of homologous genes shared between the putative acquisitions in *Mycoplasma mycoides* and representative Mycoplasmataceae species outside of the Mycoides cluster.
